# VAS: a convenient web portal for efficient integration of genomic features with millions of genetic variants

**DOI:** 10.1186/1471-2164-15-886

**Published:** 2014-10-11

**Authors:** Eric Dun Ho, Qin Cao, Sau Dan Lee, Kevin Y Yip

**Affiliations:** Department of Computer Science and Engineering, The Chinese University of Hong Kong, Shatin, New Territories, Hong Kong; Hong Kong Bioinformatics Centre, The Chinese University of Hong Kong, Shatin, New Territories, Hong Kong; CUHK-BGI Innovation Institute of Trans-omics, The Chinese University of Hong Kong, Shatin, New Territories, Hong Kong

**Keywords:** Annotation, Genetic variants, Genomic studies, Data integration

## Abstract

**Background:**

High-throughput experimental methods have fostered the systematic detection of millions of genetic variants from any human genome. To help explore the potential biological implications of these genetic variants, software tools have been previously developed for integrating various types of information about these genomic regions from multiple data sources. Most of these tools were designed either for studying a small number of variants at a time, or for local execution on powerful machines.

**Results:**

To make exploration of whole lists of genetic variants simple and accessible, we have developed a new Web-based system called VAS (Variant Annotation System, available at https://yiplab.cse.cuhk.edu.hk/vas/). It provides a large variety of information useful for studying both coding and non-coding variants, including whole-genome transcription factor binding, open chromatin and transcription data from the ENCODE consortium. By means of data compression, millions of variants can be uploaded from a client machine to the server in less than 50 megabytes of data. On the server side, our customized data integration algorithms can efficiently link millions of variants with tens of whole-genome datasets. These two enabling technologies make VAS a practical tool for annotating genetic variants from large genomic studies. We demonstrate the use of VAS in annotating genetic variants obtained from a migraine meta-analysis study and multiple data sets from the Personal Genomes Project. We also compare the running time of annotating 6.4 million SNPs of the CEU trio by VAS and another tool, showing that VAS is efficient in handling new variant lists without requiring any pre-computations.

**Conclusions:**

VAS is specially designed to handle annotation tasks with long lists of genetic variants and large numbers of annotating features efficiently. It is complementary to other existing tools with more specific aims such as evaluating the potential impacts of genetic variants in terms of disease risk. We recommend using VAS for a quick first-pass identification of potentially interesting genetic variants, to minimize the time required for other more in-depth downstream analyses.

## Background

High-density microarrays and massively parallel sequencing have made genome-wide detection of genetic variants from human DNA samples systematic, efficient and inexpensive. In these experiments, it is common to observe hundreds of thousands or even millions of loci in the DNA of a studied sample that differ from the reference genome. To explore possible links between these variants and the phenotypes of the sample, it is necessary to first analyze the potential biological significance of each variant.

Early-days analysis methods have focused on the potential impacts of genetic variants in coding regions, the functional consequences of which are usually related to alterations to the corresponding proteins. There have been many successful software tools for classifying coding variants into those that are synonymous, missense and nonsense, whether they may affect splicing or cause frameshift, and the level of disruption to protein functions and structures [1–6].

On the other hand, it is now well-recognized that many functionally important genetic variants do not change the coding sequences directly but rather perturb gene regulation [7, 8]. For example, a single nucleotide variant (SNV) may hit the binding motif of a transcription factor, which affects the proper binding of it and leads to an expression level change of the regulated gene. Since currently there is not a complete catalog of regulatory regions in the human genome, in order to determine how likely a genetic variant may affect gene regulation, one needs to examine many types of static and cell/tissue-specific features indicative of functional significance. Static features such as evolutionary conservation and sequence motifs help evaluate the possibility for a genomic region to ever play a functional role, while cell/tissue-specific features provide information about regulatory activities in each genomic region in particular cell/tissue types and conditions. Combining both types of features provides a quick and low-cost way to pinpoint the potentially most interesting variants for downstream validation and functional studies. For example, DNase I hypersensitivity and certain histone marks together could identify regulatory regions active in particular cell types that are far away from their target genes [9], while integrating such information with sequence motifs could further predict the transcription factors involved in the gene regulation.

A large amount of data containing cell/tissue-specific features have been produced for various human cell types in large-scale studies such as ENCODE [8] and Roadmap Epigenomics [10]. To utilize these data in studying genetic variants, a number of Web tools have been developed for automatic large-scale genomic data integration [11–20]. Each of them provides a database of genomic features collected from multiple data sources, and a procedure for users to query selected features around their genetic variants. These tools face two common challenges, namely 1) A list of genetic variants in standard Variant Call Format (VCF) could take up hundreds of megabytes and need a long time to upload; and 2) Integrating a long variant list with a large number of whole-genome features is time-consuming.

Concerning the data uploading issue, some tools restrict the maximum number of genetic variants per job to a small value, while others do not set an explicit limit but practically cannot handle full lists of millions of variants [11–13, 17]. Some other tools avoid the uploading of large files by allowing local installation and execution, which requires a large amount of genomic features to be downloaded to the user machine [18].

Regarding the data integration issue, most tools use a relational database to store the collected data. As a result, a table join between a stored feature and the uploaded genetic variants is often performed by time-inefficient algorithms that make use of standard tree-based indices. Although more efficient linear-time sort-merge join algorithms are available, it could be difficult to instruct the query optimizer to use them. Some tools attempted to solve this problem by pre-computing the results of a large amount of table joins [18, 21], which requires extra disk space for storing the pre-computed results and new pre-computation needs to be performed every time a new genomic feature is added to the database.

To overcome these two issues, we have developed VAS (Variant Annotation System), a tool for efficient genomic data integration.

## Implementation

**Figure 1:**
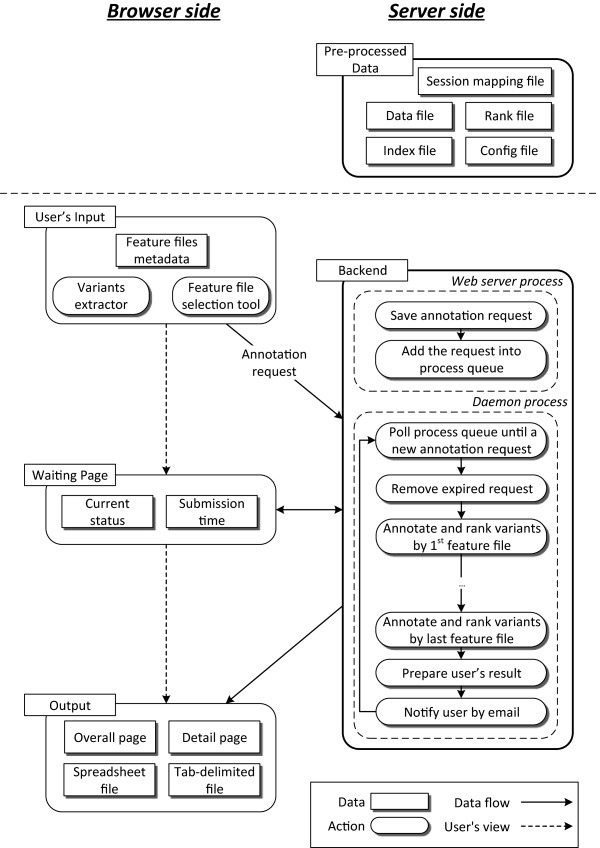
**Schematic illustration of the VAS workflow.** Genomic features are pre-sorted and stored in data files with pointers for direct access to particular genomic locations. A user supplies the list of genetic variants and selects the genomic features to integrate with the variants at the client side. The variants extractor produces a compressed form of the input variants. The task is then sent to the backend and put into a waiting queue, and the user is shown a waiting page. When an execution daemon becomes available, it fetches the next task in the queue and uses the customized algorithms to perform data integration. The integration results are stored in a tab-delimited file. The user will then be shown a summary page of the integration results. An email notification will also be sent, with a link for a user to retrieve the summary page later. The user can then view the integration details of each input variant, perform interactive analysis on the UCSC Genome Browser, or download the annotation results in tab-delimited or Excel format.

The overall workflow of VAS is shown in Figure 1. Below we describe its different components in detail.

### Genomic features in VAS

VAS provides a large variety of genomic features collected from different data sources (Table 1). To help explore genetic variants in non-coding regions, it provides a rich set of whole-genome features about sequence patterns, conservation, chromatin states and expression signals from various experimental and computational data sets. Cell/tissue-specific data based on different cell types studied by the ENCODE Project Consortium and Roadmap Epigenomics are provided for some features. Additional features are provided for referencing previous findings about known variants and their loci, including previously cataloged SNPs, information about disease SNPs, and Gencode gene annotation, which contains a large number of non-coding RNAs.

**Table 1:** **List of genomic features provided by VAS**

Type	Genomic features
Chromatin	ENCODE open chromatin, histone modifications,protein-DNA binding [8], Roadmap EpigenomicsDNA methylation [10]
Genomic states	ChromHMM segmentation [22], supervised genomicregion classification [23]
Expression	ENCODE RNA-seq [8]
Sequence	UCSC [24] conservation scores [25, 26], transcriptionfactor binding motifs [27], sequence uniqueness [28],repeats [29], GC content
Annotation	Gencode [30]
Variations	dbSNP [31]
Diseases	GWAS Catalog [32], The Human Gene MutationDatabase [33]

### Feature selection, data compression and data integration

**Figure 2:**
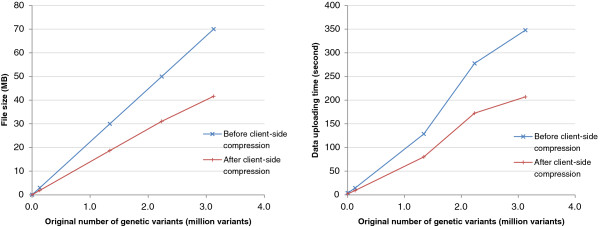
**Amount of data upload and uploading time required at various sizes of the input list of genetic variants in our simulation study, before and after client-side data compression.** The data uploading time for the uncompressed case was estimated based on the file size and the data transfer rate when transferring the compressed version of the same files.

**Figure 3:**
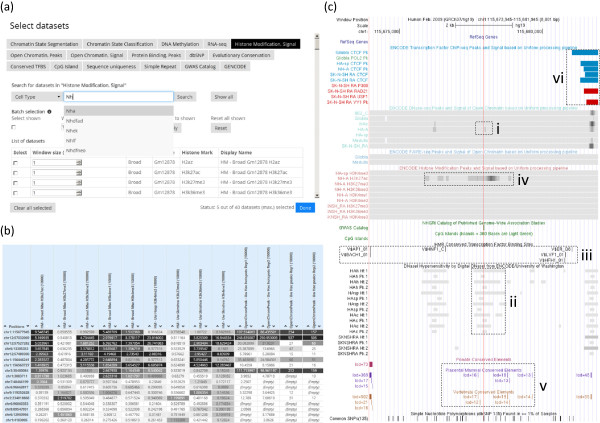
**Usage of VAS. (a)** Selecting genomic features to be integrated with the genetic variants. **(b)** Summary of the annotation results. Genomic features identified around each genetic variant (within a 10 kb window in this case) are shown, where a darker color indicates a stronger signal value. **(c)** Detailed view of a genetic variant, with an embedded UCSC Genome Browser image in which each genomic feature is shown as a signal track.

A user uploads a list of genetic variants and selects the features to be integrated through a user-friendly Web interface. Multiple data formats are supported for the input list of genetic variants, including VCF and white-space-delimited lists. In our test, uploading 3 million genetic variants involved less than 50 megabytes of data transfer (Figure 2). The enabling technology behind this small uploading data size is a compression procedure that VAS performs on the client side. In a standard VCF file, there is a lot of information not required for the data integration purpose. Our Flash plugin takes the user-supplied variant file, retains only genomic locations, and removes repetitive text such as chromosome names. The resulting file contains compact arrays of chromosomal locations, one for each chromosome. This compression process is transparent to the user in that a user only needs to specify a standard genetic variant file as input and the compression will be automatically performed before the compressed data is transferred to the server.The genomic features to be integrated with the genetic variants are selected from a Web interface that provides a list of the features available. Functions are also provided for searching for particular datasets using their attributes such as cell type (Figure 3a). For each genetic variant, VAS can search for genomic features overlapping its exact location or a flanking window of it up to 1Mb, allowing exploration of nearby loci in genetic linkage to the input variants.

Upon submitting the input variants and the selected genomic features, the data integration job is added to a queue on the server side. The back-end system adopts a scalable design that allows executing multiple jobs on different computing nodes in parallel. The user is redirected to a waiting page that provides the latest status of the job. Optionally, if an email address is entered, an email notification will be sent to the user when the job is finished.

We store data in a customized file format without relying on a relational database, which facilitated our design of linear-time integration algorithms that can efficiently identify overlapping genomic regions in different data files. Specifically, for each feature, the genomic regions containing feature values are sorted according to their genomic locations. Special pointers are added to particular locations (such as the start of each chromosome) in the genome to allow direct access of these locations without a sequential scan of all regions from the beginning of the file.

**Figure 4:**
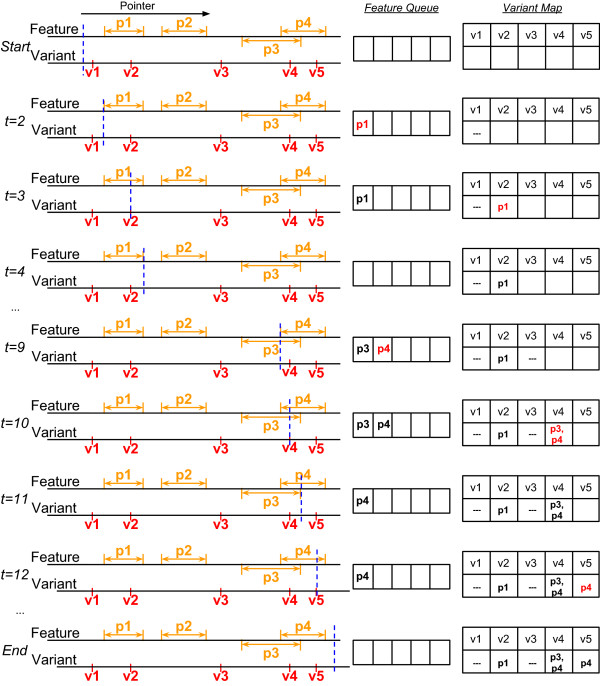
**An example of point-to-region data integration using our algorithm.**

We provide two types of data integration. The first one is identifying genomic features overlapping exactly the locations of the input genetic variants (exact location for an SNV or insertion, mid-point for a deletion). The second one is identifying genomic features overlapping a flanking window of each input genetic variant. Both types of integration are performed by sort-merge algorithms.For the first type of data integration, we first sort the input variants according to their locations. We then use a pointer to scan through all the genetic variants and all the genomic feature regions sequentially. Whenever a region of the genomic feature is encountered, we add it to a feature queue. Any genetic variant that is then encountered before the end of the region will be annotated with the region and the result is stored in the variant map (see Figure 4 for an example). More specifically, during the scanning process, the algorithm takes one of the following actions whenever a point of the corresponding type is encountered:

-  Location of a variant: Annotate the variant with all the regions currently in the feature queue and store the results in the variant map
-  Starting position of a feature region: Add the region to the feature queue
-  Ending position of a feature region: Remove the region from the feature queue

**Figure 5:**
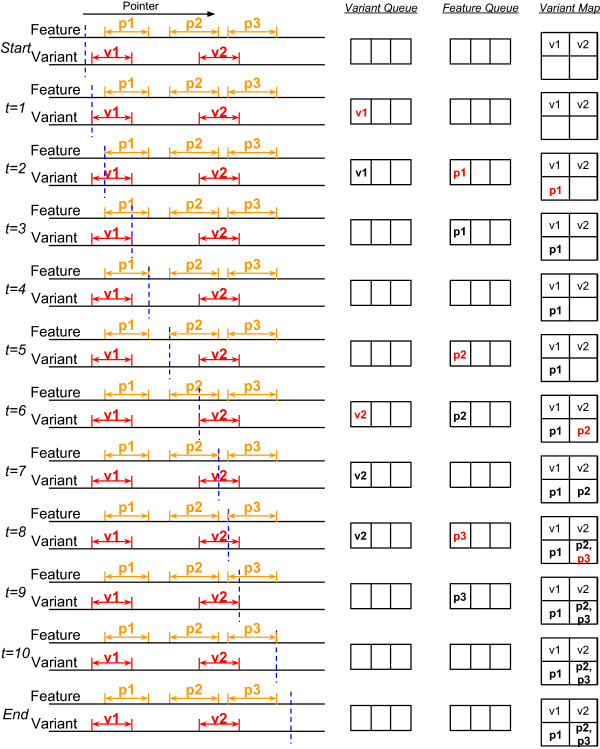
**An example of region-to-region data integration using our algorithm.**

For the second type of data integration, the integration algorithm is similar to the one for the first type, except that now instead of considering a single location of each genetic variant, we consider the starting and ending positions of its flanking window. During the scanning process, the algorithm takes one of the following actions whenever a point of the corresponding type is encountered (see Figure 5 for an example):

-  Starting position of the flanking window of a variant: Add the variant to the variant queue, annotate the variant with all the regions currently in the feature queue and store the results in the variant map
-  Ending position of the flanking window of a variant: Remove the variant from the variant queue
-  Starting position of a feature region: Add the region to the feature queue, annotate all variants currently in the variant queue with the region and store the results in the variant map
-  Ending position of a feature region: Remove the region from the feature queue

We have compared the speed efficiency of these data integration algorithms with some alternative methods. For all the methods, we tried to integrate a list of 57,902 variants with a genomic feature with 17,524 regions. We tested both types of data integration, with the size of the flanking window set to 100bp in the second type of integration. The time needed for the different methods to perform the integration task is shown in Table 2. Our customized algorithms were found to be the most efficient among the methods in comparison.When the data integration is finished, the results are displayed on a Web page that shows information about the selected features around each input variant (Figure 3b). In the case of numeric features, the average feature values around each variant and their percentiles among all genomic regions are also shown. Details of the features can be displayed in a signal-track image generated by the UCSC Genome Browser (Figure 3c). Linking to a corresponding UCSC Genome Browser session is provided for more visualization options and interactive explorations. Integration results can also be downloaded in Microsoft Excel or tab-delimited formats for further analyses.

**Table 2:** **Data integration time of different methods**

Method	Integrating variant	Integrating variant flanking
	locations (second)	windows (second)
BigBed	277.90	275.63
Interval tree	0.41	0.60
Relational database	8.05	736.23
Tabix	8.87	8.88
Our algorithms	0.21	0.52

For BigBed reader and interval tree, we used the implementation of bxpython. For relational database, we tried several indexing methods including standard B-tree index and spatial index, and report here the shortest time among these approaches. Tabix was called using the pytabix library in Python.

Each data integration job is given a unique 512-bit identifier. The user who issues a job can browse and download the results at a later time by using the provided hyperlink with this identifier embedded. All job files are kept on the server for 30 days. Other users without this identifier are unable to access the uploaded data or the corresponding data integration results.

Currently there are several related tools providing genome-wide annotation of genetic variants. Each of these tools has its unique features and advantages. We list in Table 3 some of the distinctive properties of VAS.

**Table 3:** **Some distinctive features of VAS as compared to some related tools**

Tool	CADD [16]	GEMINI [18]	GWASdb [17]	GWAVA [19]	HaploReg [20]	RegulomeDB [12]	VAS
Client-side data compression	No	(local)	N/A	No	No	No	Yes
Input variants allowed	∼100,000	(Unlimited)	1	>10,000	10,000	∼5,000	3,000,000
Genomic features/aggregated	63	(User defined)	37	14	10	1,012	1,000+
features provided			(5 categories)		(6 categories)	(13 categories)	(16 categories)
Data storage and integration	(Not described)	Relational DB	Relational DB	(Not described)	Relational DB	Relational DB	Customized
Searching flanking regions	No	No	Yes	No	No	No	Yes
Asynchronous access of results	Yes	(local)	No	No	No	No	Yes
Linkout to genome browser	No	No	UCSC [24]	Ensembl [34]	No	UCSC	UCSC

For GWAVA and RegulomeDB, the maximum number of input variants allowed is based on our own tests of the system. Properties of the tools are based on their versions on 8th September 2014.

## Results and discussion

### Case studies

As a demonstration of using VAS in exploring potential functional meanings of genetic variants, we used it to analyze two sets of genetic variants with different sets of genomic features.

The first set of genetic variants includes the susceptibility loci for migraine identified in a recent study [35]. In that study, a genome-wide meta-analysis was performed on the data from 29 genome-wide association studies, which together involved 23,285 individuals with migraine and 95,425 population-matched controls. Twelve loci were identified to be significantly associated with migraine, while 5 loci were found to have significant expression quantitative trait loci (eQTL). We used VAS to retrieve information about various types of static and cell-specific data around these 17 loci. For static features, we considered evolutionary conservation, known variants in dbSNP and GWAS Catalog, protein binding motifs and CpG islands. For cell-specific features, we considered histone modifications, open chromatin and transcription factor binding data from ENCODE sequencing experiments for both normal brain and spinal cord cells (HAc, HA-h, HA-sp and NH-A) and brain cancer lines (BE2_C, Gliobla, Medullo and SK-N-SH_RA).

Figure 3b shows part of the annotation results, where the darkness of a table entry indicates how strong the signal value is. It can be seen that many features have strong signals around the susceptibility loci. As an example, Figure 3c shows the detailed view of rs12134493 (marked by the red line), which is at position 115,479,469 (hg18)/115,677,946 (hg19) of chromosome 1. It is located in an intergenic region downstream of and close to the TSPAN2 gene. In the original study [35], it was found that the susceptibility loci in general had strong open chromatin signals in terms of DNase I hypersensitivity, and they overlapped with some transcription factor binding motifs. Consistent with their findings, VAS was able to find overlaps between the SNP and open chromatin signals in various normal brain cells (Figure 3c i, ii) and the presence of binding motifs for multiple transcription factors around that region (Figure 3c iii).We also made a number of additional interesting observations based on the VAS results. First, the open chromatin signals were found only in normal brain cells but not in the cancer line SK-N-SH_RA. Second, in astrocytes (NH-A), the SNP overlapped a local region with strong H3K27ac signals (Figure 3c iv), which suggests that the region could be an active enhancer in this cell type. Third, the SNP was inside a region with strong evolutionary conservation among placental mammals and among vertebrates (Figure 3c v), suggesting that the region is under evolutionary constraints. Finally, there was active binding of CTCF, RAD21 and YY1 in a nearby region a few kilobases away (Figure 3c vi) with corresponding open chromatin signals. Given the closeness of this region and the susceptibility locus, it may be useful to include this region into the study.

The second set of genetic variants comes from the Personal Genome Project [36] ( https://my.pgp-hms.org/). We randomly downloaded 5 lists of genetic variants with at least one variant reported to have high clinical importance according to the report on the Web site (Table 4). We tested if we could identify these variants of potential clinical importance using VAS, by annotating them with the information from GWAS Catalog [32] and the Human Gene Mutation Database [33]. On average, uploading and completing the annotation of each data file took less than 10 minutes. VAS was able to annotate all 21 unique variants reported to be likely pathogenic and rare pathogenic using the information from the two databases, which confirms that VAS can be used to quickly integrate information from diverse sources for more in-depth downstream analyses.

**Table 4:** **Lists of genetic variants from the personal genome project tested on VAS**

Sample	Total number of variants		PGP variants	Chromosomal location	dbSNP ID	Clinical importance	Found by VAS
hu47A9D1	960,613		APOA5-S19W	chr11:116662407/chr11:116167616	rs3135506	Low	Yes
			APOE-C130R	chr19:45411941/chr19:50103780	rs429358	High	Yes
			MBL2-G54D	chr10:54531235/chr10:54201240	rs1800450	Low	Yes
			MBL2-R52C	chr10:54531242/chr10:54201247	rs5030737	Low	Yes
			MTRR-I49M	chr5:7870973/chr5:7923972	rs1801394	Low	Yes
			MYO7A-R302H	chr11:76869378/chr11:76547025	rs41298135	High	Yes
			rs5186	chr3:148459988/chr3:149942677	rs5186	Low	Yes
hu7DA960	960,613		AMPD1-Q12X	chr11:115236057/chr11:115037579	rs17602729	Low	Yes
			KCNE1-D85N	chr21:35821680/chr21:34743549	N/A	High	Yes
			KRT5-G138E	chr12:52913668/chr12:51199934	rs11170164	Low	Yes
			MBL2-G54D	chr10:54531235/chr10:54201240	rs1800450	Low	Yes
			rs5186	chr3:148459988/chr3:149942677	rs5186	Low	Yes
hu8D40D6	598,897		APOE-C130R	chr19:45411941/chr19:50103780	rs429358	High	Yes
			HFE-S65C	chr6:26091185	N/A	Low	Yes
			MTRR-I49M	chr5:7870973/chr5:7923972	rs1801394	Low	Yes
			PRPH-D141Y	chr12:49689404	rs58599399	High	Yes
			RPF1-A91V	chr10:72360387/chr10:72030392	rs35947132	Low	Yes
			SERPINA1-E288V	chr14:94847262/chr14:93917014	rs17580	Low	Yes
hu998A3D	960,613		BTD-D444H	chr3:15686693/chr3:15661696	rs13078881	Low	Yes
			C3-R102G	chr19:6718387/chr19:6669386	rs2230199	Moderate	Yes
			COL4A1-Q1334H	chr13:110818598/chr13:109616598	rs3742207	Low	Yes
			HFE-S65C	chr6:26091185	N/A	Low	Yes
			MTRR-I49M	chr5:7870973/chr5:7923972	rs1801394	Low	Yes
			rs5186	chr3:148459988/chr3:149942677	rs5186	Low	Yes
			SERPINA1-E366K	chr14:94844947/chr14:93914699	rs28929474	High	Yes
hgD53911	612,647		COL4A1-Q1334H	chr13:110818598/chr13:109616598	rs3742207	Low	Yes
			MTRR-I49M	chr5:7870973/chr5:7923972	rs1801394	Low	Yes
			PKD1-R4276W	chr16:2139814/chr16:2079814	rs114251396	High	Yes
			rs5186	chr3:148459988/chr3:149942677	rs5186	Low	Yes
			SCNN1G-E197K	chr16:23200963/chr16:23108463	rs5738	Low	Yes
			VWF-R854Q	chr12:6143978/chr12:6014238	rs41276738	Moderate	Yes

The variants listed in the "PGP variants" column include likely pathogenic and rare (<2.5%) pathogenic variants according to the reports available on the Personal Genome Project Web site. The information in the "Chromosomal location", "dbSNP ID" and "Clinical importance" columns was all obtained from these reports.

### Data uploading and integration time

To test the speed performance of VAS in handling large data files, we recorded the time required to integrate 6.4 million genetic variants present in the CEU trio obtained from the 1000 Genomes Project with the information of the whole list of SNPs in dbSNP. We compared the performance of VAS with both the reported results and our local execution of GEMINI [18], a tool that allows large-scale genomic data integration by means of local execution and pre-caching of table join results. Both VAS and our local execution of GEMINI were tested on a machine with dual quad core Xeon CPU at 2.4 GHz and 64 GB of main memory.

The resulting time measurements of the two tools (Table 5) show that VAS finished the data integration in around half an hour. As for GEMINI, although our time measurements are different from those reported in the original paper due to the use of different machines, in general a long data loading time (1.5–3 hours) was required for the extensive pre-computation, followed by a very quick data integration phase. This pre-computation step needs to be performed whenever a new set of genetic variants is to be annotated.Since GEMINI was executed locally while VAS is an online system, there was extra data uploading time for VAS. For the data set tested, the data uploading time was negligible as compared to the time needed for data integration. This result is consistent with our above analysis on file size and data uploading time at different numbers of input genetic variants (Figure 2).

**Table 5:** **Time measurement of GEMINI and VAS**

Tool		Data loading/uploading (s)*	Data integration (s)	Total (s)
**GEMINI (as reported in** [18]**)**	**Average**	**5,050.0**	**24.0**	**5,064.0**
GEMINI (our testing results)	Trial 1	9,944.6	154.1	10,098.6
	Trial 2	9,960.5	155.5	10,116.1
	Trial 3	10,182.4	156.9	10,339.3
	Trial 4	10,182.3	162.8	10,345.1
	Trial 5	10,053.2	169.1	10,222.2
	Average	10,064.6	159.7	10,224.3
	Std. dev.	115.2	6.2	117.6
VAS	Trial 1	9.9	1,711.1	1,721.1
	Trial 2	10.4	1,772.3	1,782.7
	Trial 3	9.7	1,552.5	1,562.1
	Trial 4	9.2	1,541.6	1,550.8
	Trial 5	9.6	1,580.9	1,590.5
	Average	9.8	1,631.7	1,641.4
	Std. dev.	0.4	103.7	104.1

^*^Time for GEMINI to load the data into database and perform pre-computations, and time for VAS to upload the file from the client browser to our server.

Overall, VAS is more efficient and flexible in handling new variant lists since it does not require pre-loading of data, while GEMINI works better in situations where the same list of genetic variants is to be repeatedly analyzed by integrating with many different subsets of genomic data.

## Conclusion

In this paper, we have described VAS, a new Web tool that can efficiently integrate millions of genetic variants with tens of whole-genome data sets in a single integration task. The client-side data compression procedure and the customized data store allowed fast uploading and integrating whole lists of genetic variants obtained from genomic studies, making VAS a practical tool for routine first-step annotation of genetic variants.

When analyzing large-scale genomic data, the main bottleneck is usually inspecting long lists of results, pinpointing the most biologically or medically significant parts, and making correct interpretations of them. The time spent on data integration is usually relatively unimportant. However, the time difference between a standard data integration method and a customized one could become large when the numbers of input genetic variants and integrating genomic features are large. In addition, since VAS can accept multiple job requests from different users simultaneously, having an efficient data integration method can also shorten the time spent on waiting for other earlier jobs in the queue to complete.

Currently VAS supports job-level parallelization, which means multiple jobs can be run at the same time in parallel on different computing units. In the future, we plan to extend VAS to support sub-job-level parallelization, which means a single job can be divided into sub-tasks simultaneously performed on different computing units. As the integration of each genetic variant is independent of the other variants, high-level distributed computing frameworks such as MapReduce should be readily applicable. An additional advantage of adopting such a framework is the distribution of data to multiple machines, which allows for better scalability.

VAS is currently implemented as an online system, which enjoys the advantage of requiring no local installation or downloading of genomic features by the user. We have ensured data integrity and confidentiality by providing encrypted network connections and assigning task IDs that are only made known to the users who submit the tasks. However, there are situations in which some private data can only be analyzed locally. Theoretically a user can install a local version of VAS on his/her own machine to perform the analysis offline, but that would also require downloading a large amount of stored data features. We will investigate ways to facilitate data integration in these situations, such as allowing users to easily download a selected subset of features or dynamically download data features at the time needed, and developing privacy-preserving distributed data integration algorithms.

In the case study we have demonstrated that with the data currently loaded into VAS, one could already use it to obtain some interesting patterns around each genetic variant. As more and more cell/tissue-specific data are being produced, we will keep updating the data repository of VAS to cover more cell/tissue types and more data for each cell/tissue type. We also plan on supporting the GRCh38 human reference genome when most data files in our database have a CRCh38 version available.

## Availability and requirements

**Project name:** Variant Annotation System (VAS)

**Project home page:**https://yiplab.cse.cuhk.edu.hk/vas/

**Operating system:** VAS can be accessed from any platform by using one of the listed Web browsers

**Programming languages:** PHP, Python

**Other requirements:** We recommend accessing VAS by using Google Chrome (version 35 or higher), Microsoft Internet Explorer (version 10 or higher), or Mozilla Firefox (version 24 or higher), with JavaScript enabled and a minimum screen resolution of 1024 pixels x 768 pixels

**Any restrictions to use by non-academics:** Nil
